# Nanochitosan antimicrobial activity against *Streptococcus mutans* and *Candida albicans* dual-species biofilms

**DOI:** 10.1186/s13104-019-4422-x

**Published:** 2019-07-08

**Authors:** Radyum Ikono, Agnia Vibriani, Indra Wibowo, Kurniawan Eko Saputro, Wibias Muliawan, Boy Muchlis Bachtiar, Etik Mardliyati, Endang Winiati Bachtiar, Nurul Taufiqu Rochman, Hideaki Kagami, Li Xianqi, Tokiko Nagamura-Inoue, Arinobu Tojo

**Affiliations:** 1 Division of Bionanotechnology, Nano Center Indonesia, Tangerang Selatan, Indonesia; 2 Department of Metallurgical Engineering, Sumbawa University of Technology, Sumbawa Besar, Indonesia; 3 Division of Molecular Therapy, The Institute of Medical Science, University of Tokyo, Tokyo, Japan; 4 School of Life Science and Technology, Bandung Institute of Technology, Bandung, Indonesia; 5 Oral Science Laboratory, Department of Oral Biology, Faculty of Dentistry, Universitas Indonesia, Jakarta, Indonesia; 6 Center for Pharmaceutical and Medical Technology, Agency for the Assessment and Application of Technology [BPPT], Tangerang Selatan, Indonesia; 7 Research Center for Physics, Indonesian Institute of Science [LIPI], Tangerang Selatan, Indonesia; 8 Department of Oral and Maxillofacial Surgery, Matsumoto Dental University, Shiojiri, Japan; 9 Department of General Medicine, IMSUT Hospital, The Institute of Medical Science, University of Tokyo, Tokyo, Japan; 10 Department of Cell Processing and Transfusion, The Institute of Medical Science, University of Tokyo, Tokyo, Japan

**Keywords:** Biofilm, *Candida albicans*, Caries, Nanochitosan, *Streptococcus mutans*

## Abstract

**Objective:**

Chitosan nanoparticle (nanochitosan) has a broad antimicrobial spectrum against diverse pathogenic microorganisms. However, its effect on dental caries-associated microorganisms, such as *Streptococcus mutans* and *Candida albicans* is yet to be explored. These microorganisms are known for causing early childhood caries. Therefore, this study was aimed at investigating nanochitosan inhibition capacity against dual-species biofilms of *S. mutans* and *C. albicans.* In this study, nanochitosan antimicrobial activity is reported against mono and dual biofilm species of *S. mutans* and/or *C. albicans* at 3 and 18 h incubation time. Nanochitosan inhibition capacity was observed through biofilm mass quantity and cell viability.

**Results:**

The present study successfully synthesized nanochitosan with average diameter of approximately 20–30 nm, and also established dual-species biofilms of *S. mutans* and *C. albicans* in vitro. With nanochitosan treatment, the cell viability of both microorganisms significantly decreased with the increasing concentration of nanochitosan. There was no significant decrease in biofilm mass both in the dual and single-species biofilms after 3 h of incubation. However, greater inhibition of biofilm was observed at 18 h incubation.

## Introduction

Early childhood caries (ECC) is an aggressive form of dental caries which affects most children (< 72 months age) in developing countries. In South East Asia region, the prevalence is reported to be in ranges of 25% to 95% [3]. The onset of ECC starts with formation of biofilms from cariogenic microorganisms, dominated by *Streptococcus mutans* and *Candida albicans*. Microorganisms interact synergistically in forming dual-species biofilms [1–7]. Previous work showed that glucosyltransferase (GTF) enzymes produced by *S. mutans* could bind to the surface of *C. albicans* cells, making *C. albicans* produce glucan as a component of extracellular polymeric substances (EPS). This interaction significantly increases EPS formation and enhances antimicrobial drug tolerance in dual-species biofilms, leading to dental caries aggressive form in human and rodent model [2].

Treatment for oral biofilm-associated disease is complicated because of its multifactorial etiology. Moreover, biofilms are composed of > 90% EPS which make biofilms more resistant to antimicrobial substances owing to their limited diffusion to reach microorganism cells [8, 9]. One of treatments that have been investigated widely is by using nanoparticles. Nanoparticles are proven to have superior penetration ability, effective antimicrobial activity [8, 9], and cost effective, compared to treatment with naturally derived anti-biofilm agents [10, 11].

Chitosan is a polymer comprised of β-(1-4)-linked d-glucosamine and *N*-acetyl-d-glucosamine which possesses superior properties: antimicrobial, biocompatible, and low toxicity [12–22]. Several reports examined nanochitosan antimicrobial activity against single-species biofilms in either *Streptococcus mutans* [23] or *C. albicans* [24] only; however its effect against both dual-species biofilms have yet to be reported elsewhere. This study was therefore conducted to evaluate the potential ability of nanochitosan as an antimicrobial agent against synergism of *S. mutans* and *C. albicans* biofilms.

## Main text

### Materials and methods

#### Preparation of nanochitosan solution

Chitosan nanoparticles were prepared by ionic gelation with tripolyphosphate (TPP) crosslinking as described previously [25], with slight modifications. Then, transmission electron microscopy (JEM-2100 TEM, JEOL, Tokyo, Japan) was used to characterize the average particle size and morphology of the chitosan nanoparticles.

#### Microorganism strains and culture conditions

*Streptococcus mutans* ATCC 25175 and *Candida albicans* ATCC 10231 strains were used. Both microorganisms were cultured separately in a medium triptic soy broth (TSB) (Oxoid Limited, Hampshire, UK) supplemented with 1% sucrose (Himedia Laboratories, Mumbai, India) for 18 h at 37 °C. *Streptococcus mutans* was cultured anaerobically (10% CO_2_, 80% N_2_, 10% H_2_) and *Candida albicans* was cultured aerobically [4].

#### Saliva coating

The study obtained ethical approval from the ethical research committee of Faculty of Dentistry, University of Indonesia (no. 24/Ethical Approval/FKGUI/UI/2017). Unstimulated saliva from one healthy person was collected then centrifuged at 3000 rpm for 10 min at 4 °C. The supernatant was taken and sterilized using a 0.22 μm filter. The total protein concentration of saliva was then measured using a Qubit Protein Assay Kit (Thermo Fischer Scientific, Massachusetts, USA) and diluted using PBS (Phosphate Buffer Saline) to reach a concentration of 200 ng/mL. Subsequently, 200 µL of sterilized saliva were added to the 96-well plate and incubated for 1 h at 37 °C, then the unattached salivary protein on each well was removed [26].

#### Biofilm formation

The microorganisms which had been cultured for 18 h were harvested by centrifugation (5000 rpm, 10 min, 4 °C). Each pellet was diluted using TSB + 1% sucrose, up to OD600 = 0.1, measured using a UV/VIS spectrophotometer (SP-8001, Metertech Inc., Taipei, Taiwan).

In the dual-species biofilms group, equal volumes (100 µL) of each microorganism were inoculated into the wells. Besides, for the single-species biofilms, 200 µL of one microorganism suspensions (*S. mutans* or *C. albicans*) were inoculated on each well. Next, dual-species biofilms and single-species biofilms well-plate were incubated for 90 min under anaerobic condition (10% CO_2_, 80% N_2_, 10% H_2_).

After 90 min, supernatant was removed and replaced with 200 µL of different nanochitosan concentrations: 0% (control group), 15%, 30%, and 45%. The nanochitosan solution was diluted with TSB + 1% sucrose medium to obtain appropriate concentration. Then, well plate were re-incubated anaerobically (10% CO_2_, 80% N_2_, 10% H_2_) at different incubation time: 3 h and 18 h. After incubation, the supernatant was aspirated and the wells were washed with PBS two times. Each experiment was carried out in triplicate [4, 27, 28].

#### Biofilm mass quantification

Biofilm mass quantification was carried out using a 0.1% (v/v) crystal violet. The absorbance value was measured using a microplate reader (M965+, Metertech Inc., Taipei, Taiwan), at 600 nm. The percentage of biofilm mass remaining was calculated by the following formula [5, 28]:

$$\frac{A600 \;nanochitosan \;group}{A600 \;control \;group} \times 100$$

While the percentage biofilm mass reduction was calculated by the following formula:

$$\frac{A600\; control \;group - A600 \;nanochitosan \;group}{A600 \;control\; group} \times 100$$

The control group in the formula above refers to 0% nanochitosan group in experiment.

#### Analysis of viability of *S. mutans* and *C. albicans*

The biofilms that have been formed were scraped off from each well and put into a microtube filled with 200 µL TSB medium + 1% sucrose to obtain biofilm suspension. It was then gradually diluted (1:100) using PBS until 10^−6^ for counting *S. mutans* and 10^−4^ for counting *C. albicans*. *S. mutans* from single-species biofilm suspension were grown in the Brain Heart Infusion agar (Himedia Laboratories, India) at 37 °C anaerobically, and *C. albicans* from single-species biofilm suspension were incubated on Saboraud Dextrose agar (Oxoid Limited, Hampshire, UK) at 37 °C aerobically. Dual-species biofilms suspension were incubated both on BHI agar and SD agar. Colonies were counted in the day after [5].

#### Statistical analysis

The statistical analysis for co-colonization of *S. mutans* and *C. albicans* data was performed using Independent *t* test to compare the dual-species and single-species biofilms. Differences in viability, the remaining biofilm mass, and biofilm mass reduction among concentration of nanochitosan groups was determined using ANOVA One Way test followed by Multiple Comparison Tukey’s HSD test. The log transformation data was performed for viability of *S. mutans* and *C. albicans* before ANOVA One Way analysis. A value of *p*-value< 0.05 was considered statistically significant. All statistical analysis procedures were performed using the SPSS software package, Version 16.0 (IBM Corp., NY, USA).

### Results

#### The morphological analysis of nanochitosan

**Fig. 1:**
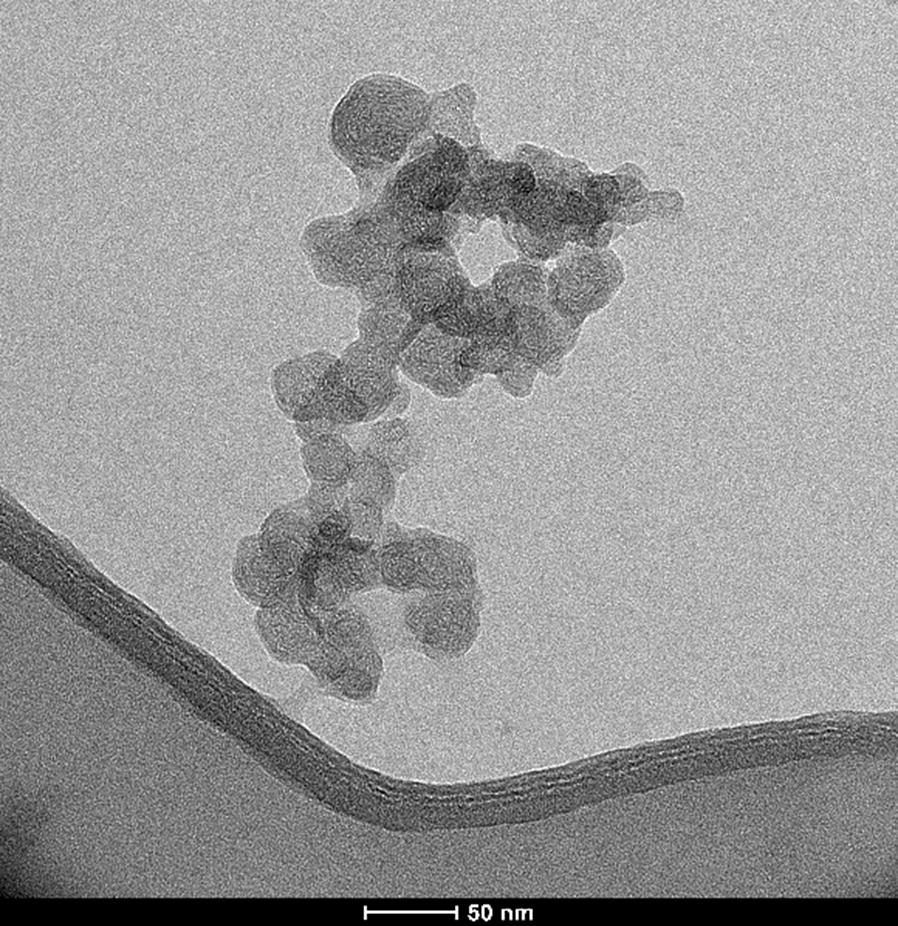
The TEM image of chitosan nanoparticles (scale bar 50 nm). This figure shows that nanochitosan has the particle size below 50 nm

Figure 1 depicts the image produced by transmission electron microscopy (TEM) of nanochitosan. The diameter of synthesized chitosan nanosphere ranged between 20 and 30 nm. The morphology and surface appearance of synthesized nanochitosan was a nearly spherical shape with a smooth surface.

#### Streptococcus mutans and *Candida albicans* co-colonization

**Fig. 2:**
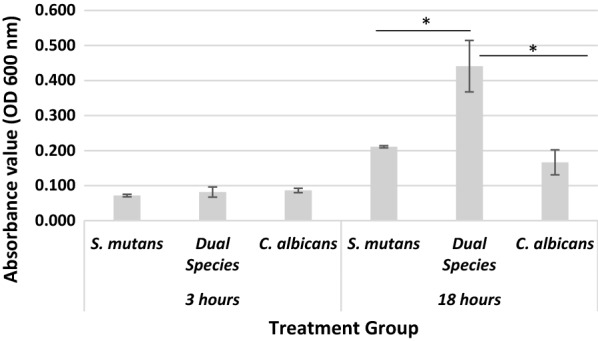
Absorbance values that show the biofilm mass of each treatment group. An asterisk mark represents significant differences between absorbance value in dual-species and single-species

The co-colonization between *S. mutans* and *C. albicans* was evaluated from biofilm mass percentage in control group. Figure 2 shows the biofilm mass based on absorbance value which represents cells and extracellular matrix. At 3-h incubation, there was no significant increase in biofilm mass both in the dual and single-species biofilms. However, at 18-h incubation, the dual-species biofilms mass was significantly higher than the single-species biofilm mass of *C. albicans* and *S. mutans.* Another important key point is, the biofilm formation grew more massively in dual-species group that was only started with half of the microbes, compared to those of single-species group.

#### Evaluation of nanochitosan antimicrobial activity on dual-species biofilms

**Fig. 3:**
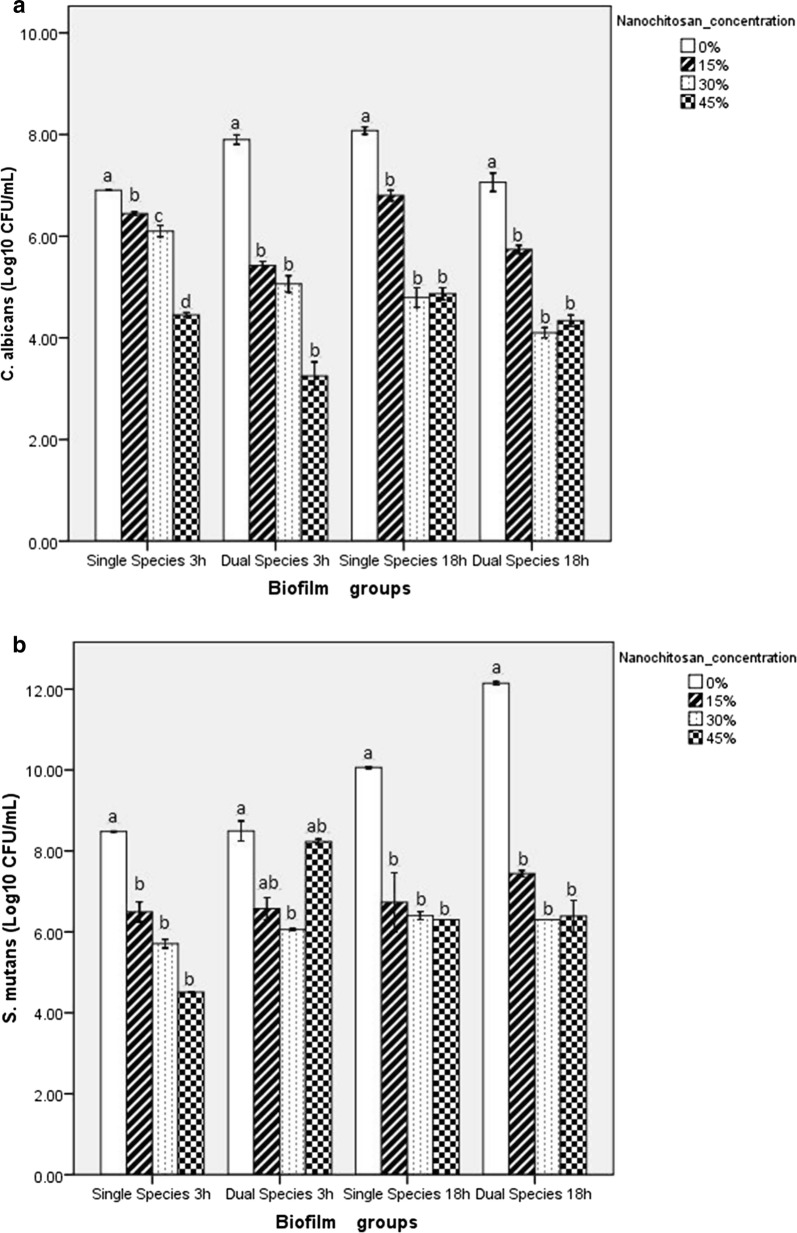

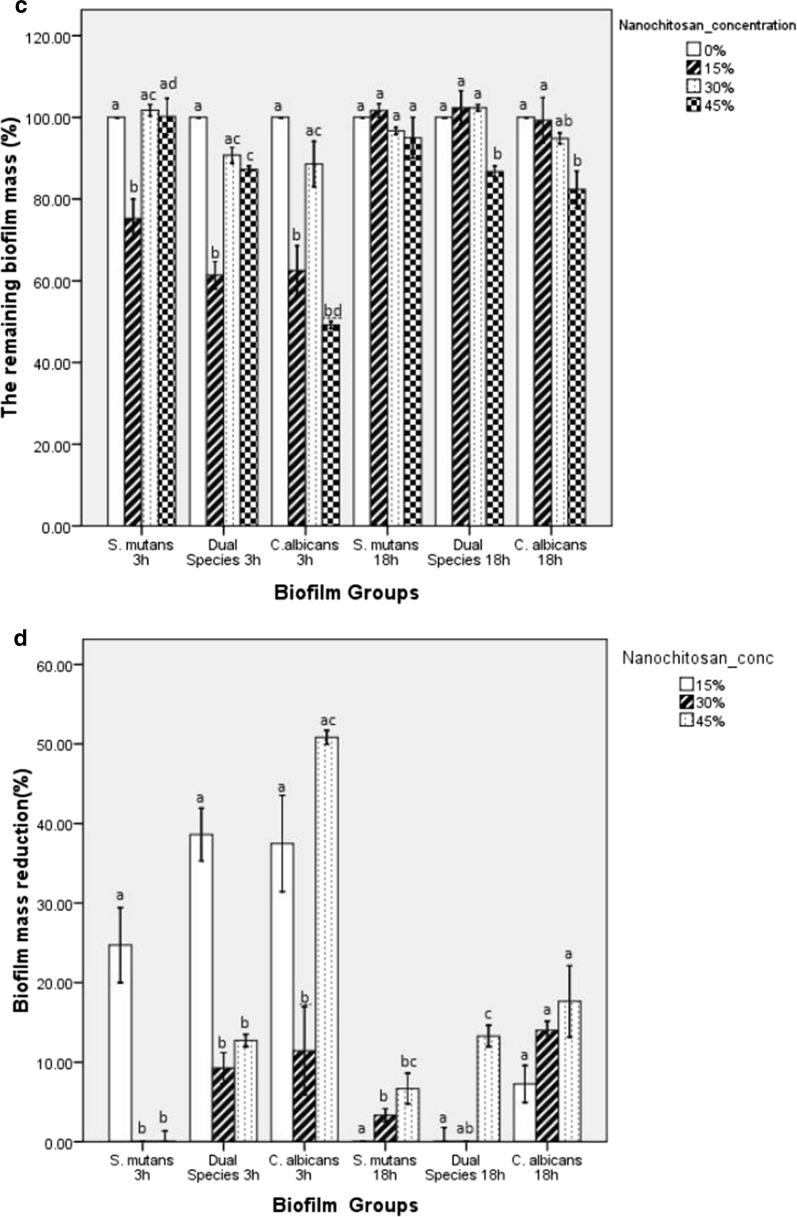
The viability of *C. albicans* (**a**) and *S. mutans* (**b**), the remaining biofilm mass (**c**) and biofilm mass reduction (**d**) in each treatment group. Bar which is designated as *S. mutans* and *C. albicans* means single-species biofilm group. Bars with different letters represent significantly different according to Tukey’s HSD test

The effect of nanochitosan on dual-species biofilms was investigated through cell viability and biofilm mass changes. Figure 3a, b represents the cell viability of *C. albicans* and *S. mutans* respectively, after treatment with nanochitosan at various concentrations. The cell viability of both species significantly decreased at 3-h and 18-h incubation, along with the increasing nanochitosan concentration.

Nanochitosan inhibition capacity was further confirmed by observing biofilm mass changes. Figure 3c shows that the remaining biofilm mass in single and dual-species at 3-h incubation increased along with the increasing nanochitosan concentration. Only at 18-h incubation, it started to show a decreasing trend of remaining biofilm mass, along with the increasing nanochitosan concentration. To strengthen the inhibition data, biofilm mass reduction was also evaluated (Fig. 3d). At 3-h incubation time, no specific trend was observed. An increase in percentage of biofilm mass reduction started to be seen with the increase in concentration of nanochitosan used at 18-h incubation.

### Discussion

The results of present study reflect the role of nanochitosan in inhibiting *C. albicans* and *S. mutans* as dual-species biofilms. We observed nanochitosan activity at 3-h and 18-h incubation against biofilms. Three hours incubation is the time required for cell surface adhesion, where production of extracellular matrix is still low. Furthermore, as bacteria and fungi continued to form mature biofilms, the quantity of extracellular matrix increased significantly. This phenomenon can be observed by measuring absorbance value as depicted in Fig. 2, where there was an increase absorbance value from 3 to 18 h, especially for the dual-species biofilms mass which was significantly higher than single-species biofilm group [2, 29, 30].

Nanochitosan inhibition capability was evaluated by measuring: cell viability, remaining biofilm mass, and biofilm mass reduction in dual-species biofilms after treating with nanochitosan at various concentrations. It has been previously studied [31, 32] that nanochitosan can inhibit the viability of biofilm cells with the effectivity of more than 90%. Accordingly, the novelty of this study stands on the effect of nanochitosan on dual-species biofilms. Dual-species biofilms are more resistant to antimicrobial agent because the interactions that occur can affect development, function, and structure of the biofilms formed; different from those in the single-species biofilm. One of the reasons is because dual-species biofilms produce more extracellular matrix than single-species biofilms, which causes limited diffusion of antimicrobial substances to reach microbial cells. As a nanoparticle, chitosan has higher penetration rate rather than other antimicrobial agents (micro size). After penetrating extracellular matrix of biofilms, nanochitosan as a cationic molecule will interact with anionic particles on the cell surface of microorganisms. Modes of action of nanochitosan as cationic biocide are adsorption on microorganism cells, diffusion through the cell wall, adsorption and destruction of the plasma membrane, cytoplasmic component leakage and cell death [33, 34]. Hence, we observed the *S. mutans* and *C. albicans* cell viability reduction comparing the treatment groups (15%, 30%, 45% nanochitosan concentration) with the control group (0% nanochitosan) (Fig. 3a, b).

However, the percentage of biofilm mass at 3-h incubation tends to increase along with the increasing nanochitosan concentration (Fig. 3c). This could be due to the fact that *S. mutans* enhance insoluble glucan synthesis by up-regulating glucosyltransferase B (*gtfB*) and glucosyltransferase B (*gtfC*) genes, as an initial response to lower pH [35]. Moreover, after reaching its maturation stage after 18-h incubation, the production of the extracellular matrix should be more stable and more numerous, yet as expected, there is an interference from nanochitosan resulting in a decrease in the percentage of biofilm mass [2, 36]. In all cases, the nanochitosan showed minimal inhibition capability against extracellular matrix disruption as shown in Fig. 3c, d, while the remaining biofilm mass was still high.

The results of the present study demonstrated that as little as 15% (v/v) nanochitosan exhibited prominent antimicrobial activity on dual-species of *S. mutans* and *C. albicans* biofilms by decreasing survival rate of microbial cells. Our experiments suggest that nanochitosan could potentially be developed as an oral-health care product, such as toothpaste and mouthwash.

## Limitations

A limitation of this experiment is that we did not assess minimal inhibitory concentration (MIC) of nanochitosan against dual-species biofilms of *Streptococcus mutans* and *Candida albicans.*

## Data Availability

The datasets used and/or analyzed during the current study are available from the corresponding author on reasonable request.

## Abbreviations

ECC: early childhood caries
EPS: extracellular polymeric substances
GtfB: glucosyltransferase B
GtfC: glucosyltransferase C
GTF: glucosyltransferase
Nanochitosan: chitosan nanoparticle
PBS: phosphate buffer saline
TEM: transmission electron microscopy
TPP: tripolyphosphate
TSB: triptic soy broth
